# 2,6-Di­bromo-3,4,5-tri­meth­oxy­benzoic acid

**DOI:** 10.1107/S2056989023007831

**Published:** 2023-09-14

**Authors:** Florian Meurer, Tanya Dimova, Michael Bodensteiner, Iliyan Kolev

**Affiliations:** a Faculty of Chemistry and Pharmacy, University of Regensburg, Universitaetsstr. 31, 93053 Regensburg, Germany; b Faculty of Pharmacy, Department of Pharmaceutical Chemistry, Medical University "Prof. Dr. Paraskev Stoyanov" Varna, 84 "Tzar Osvoboditel" Blvd., 9000 Varna, Bulgaria; Texas A & M University, USA

**Keywords:** crystal structure, Hirshfeld atom refinement (HAR), *NoSpherA2*, substituted tri­meth­oxy­benzoic acid

## Abstract

The title compound, 2,6-di­bromo-3,4,5-tri­meth­oxy­benzoic acid (DBrTMBA), C_10_H_10_Br_2_O_5_, was obtained by bromination and transhalogenation of 2-iodo-3,4,5-tri­meth­oxy­benzoic acid with KBrO_3_. Like the previously reported 2,6-di­iodo-3,4,5-tri­meth­oxy­benzoic acid (DITMBA), the structure of the title compound features a catemeric arrangement of DBrTMBA mol­ecules along an endless chain of carb­oxy­lic H–carbonyl inter­actions. A short carbon­yl–phenyl contact hints at a possible lone pair(O)–π-hole inter­action further stabilizing the chain-like structure over a dimeric arrangement of the carb­oxy­lic acid.

## Abstract 2

The previously unknown crystal structure of 2,6-di­bromo-3,4,5-tri­meth­oxy­benzoic acid was determined employing state-of-the-art Hirshfeld atom refinement and the crystal packing was analysed using Hirshfeld surface analysis.

## 1. Chemical context

Organobromine compounds are valuable precursors in organic and pharmaceutical synthesis. Their participation in homo- and cross-coupling reactions is undisputed and even preferred over the other halogen-containing compounds. In practice, many brominating agents are used for their synthesis, though few of them appear to be safe both for the user-chemist and environment. Therefore, in the present work, we present a new environmentally friendly method for the synthesis of 2,6-di­bromo-3,4,5-tri­meth­oxy­benzoic acid. Its structure is closely related to those of mono- and di­iodo-3,4,5-tri­meth­oxy­benzoic acids ITMBA and DITMBA (Kolev *et al.*, 2021 ▸, 2023 ▸). 
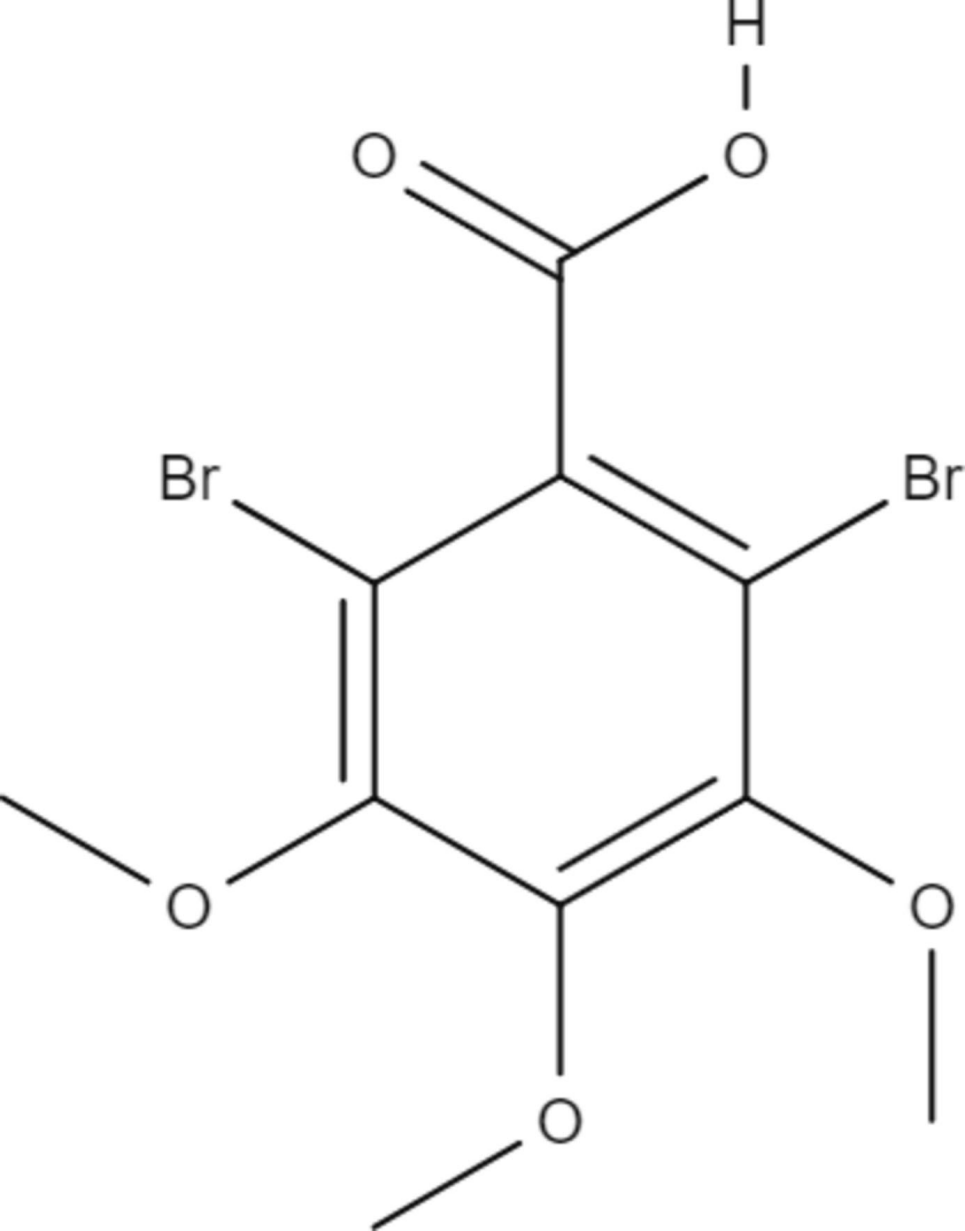


## 2. Structural commentary

**Figure 1:**
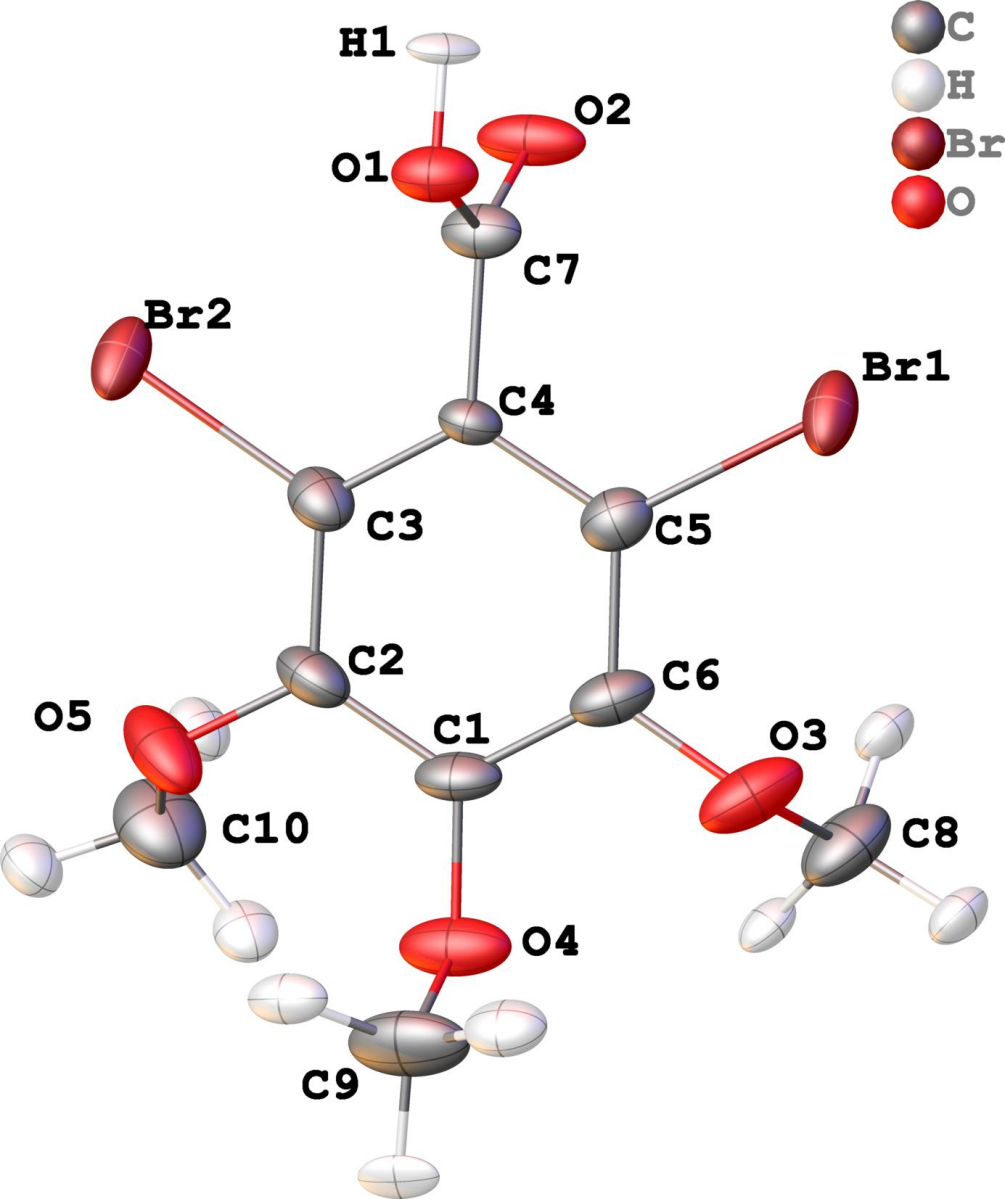
Labelling scheme and structure of DBrTMBA. Displacement ellipsoids are drawn at the 50% probability level.

DBrTMBA (Fig. 1 ▸) crystallizes in the monoclinic space group *P*2­_1_ */n* with one acid mol­ecule in the asymmetric unit (*Z* = 4). The carb­oxy­lic acid (O1/C7/O2) group is almost perpendicular to the geometrical C_6_ mean plane and at an angle of 86.7 (2)°. This derivation is exactly in the middle of the reported geometries of the catemeric DITMBA, which is closer to 90° and the reported dimeric DITMBA·toluene, which deviates more from 90° (Kolev *et al.*, 2023 ▸).

## 3. Supra­molecular features

**Figure 2:**
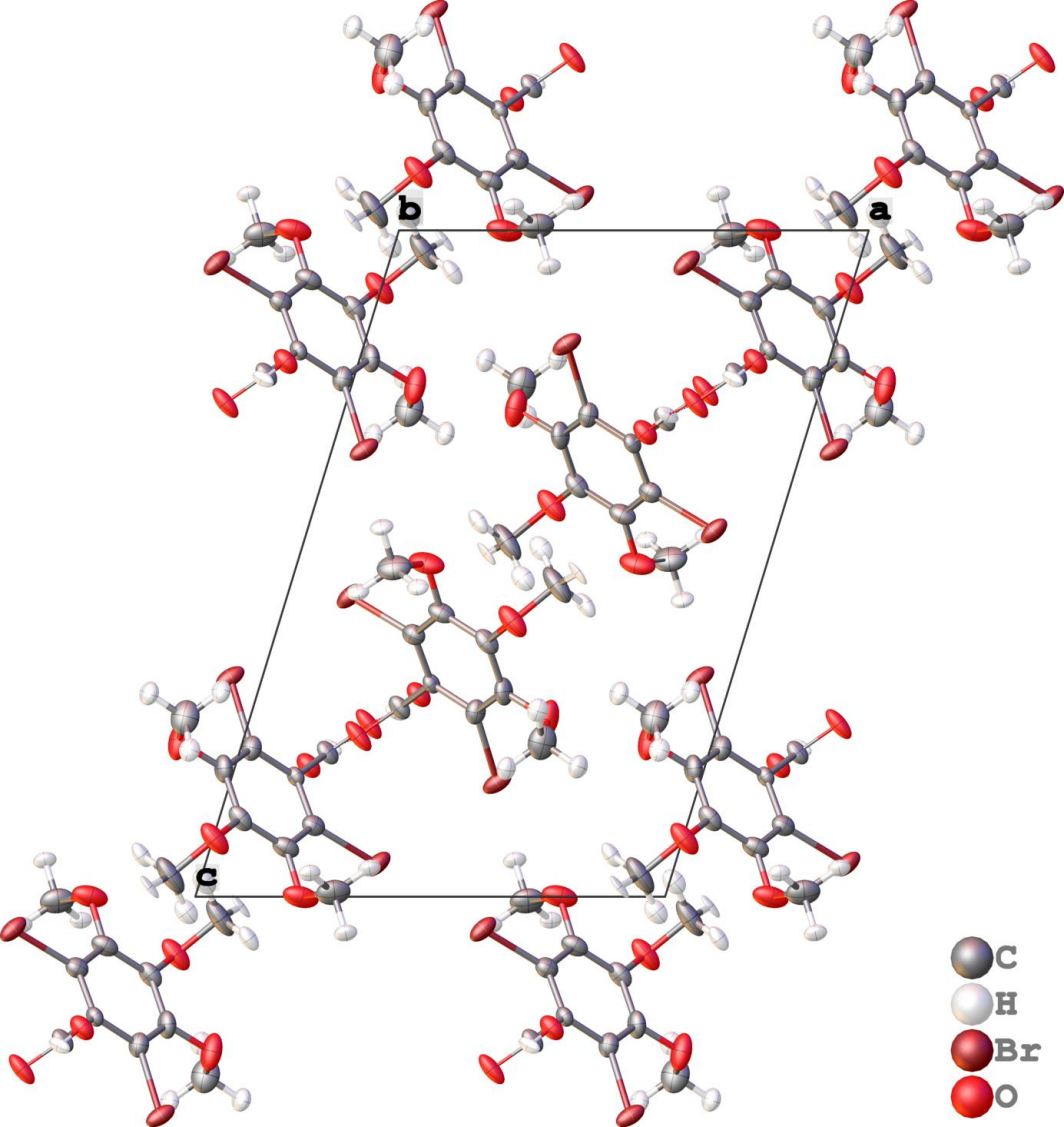
Packing of DBrTMBA along the crystallographic *b*-axis direction.

**Figure 3:**
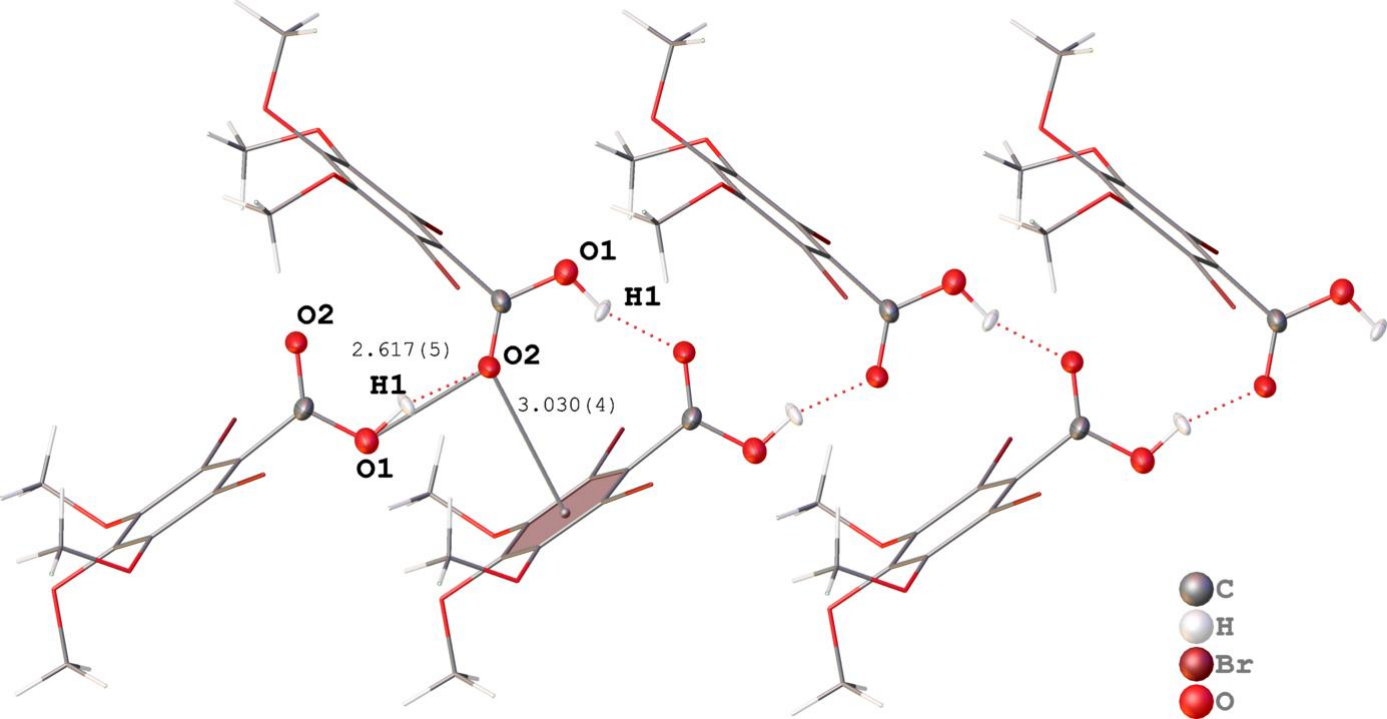
Syndiotactic arrangement of DBrTMBA in the crystallographic *b*-axis direction with O(H)—O and O–center of gravity C_6_ and distances in Å. Atoms that are not part of the carb­oxy­lic group are shown in stick representation for clarity.

Different to the also related structures of mono- and di­iodo-3,4,5-tri­meth­oxy­benzoic acids ITMBA and DITMBA·toluene (Kolev *et al.*, 2021 ▸, 2023 ▸), the title compound exhibits no dimeric structure in the solid state (Fig. 2 ▸). Instead, a hydrogen-bonded chain along the crystallographic *b*-axis direction between neighbouring acids is observed. Mol­ecules of DBrTMBA are arranged in a catemeric fashion along this chain of carb­oxy­lic hydrogen inter­action. The structure is thus very similar to that of solvent-free DITMBA (Kolev *et al.*, 2023 ▸). The O1—O2 distance of the DBrTMBA inter­molecular hydrogen-bonding inter­action is 2.617 (5) Å (Table 1 ▸, Fig. 3 ▸).

Another inter­esting structural feature in this syndiotactic arrangement can be described as a carbonyl O2 lone pair(lp)–π (C_6_) contact with a distance from O2 to the center of geometry of the benzene ring of 3.030 (4) Å (Fig. 2 ▸). This contact presumably contributes to the deviation from the dimeric structure as observed in ITMBA and DITMBA· toluene (Kolev *et al.*, 2021 ▸, 2023 ▸). In the latter, the toluene solvent mol­ecule seems to shield the C_6_ π system from this kind of inter­action, giving rise to a preferred dimeric structure in this solvate.

**Figure 4:**
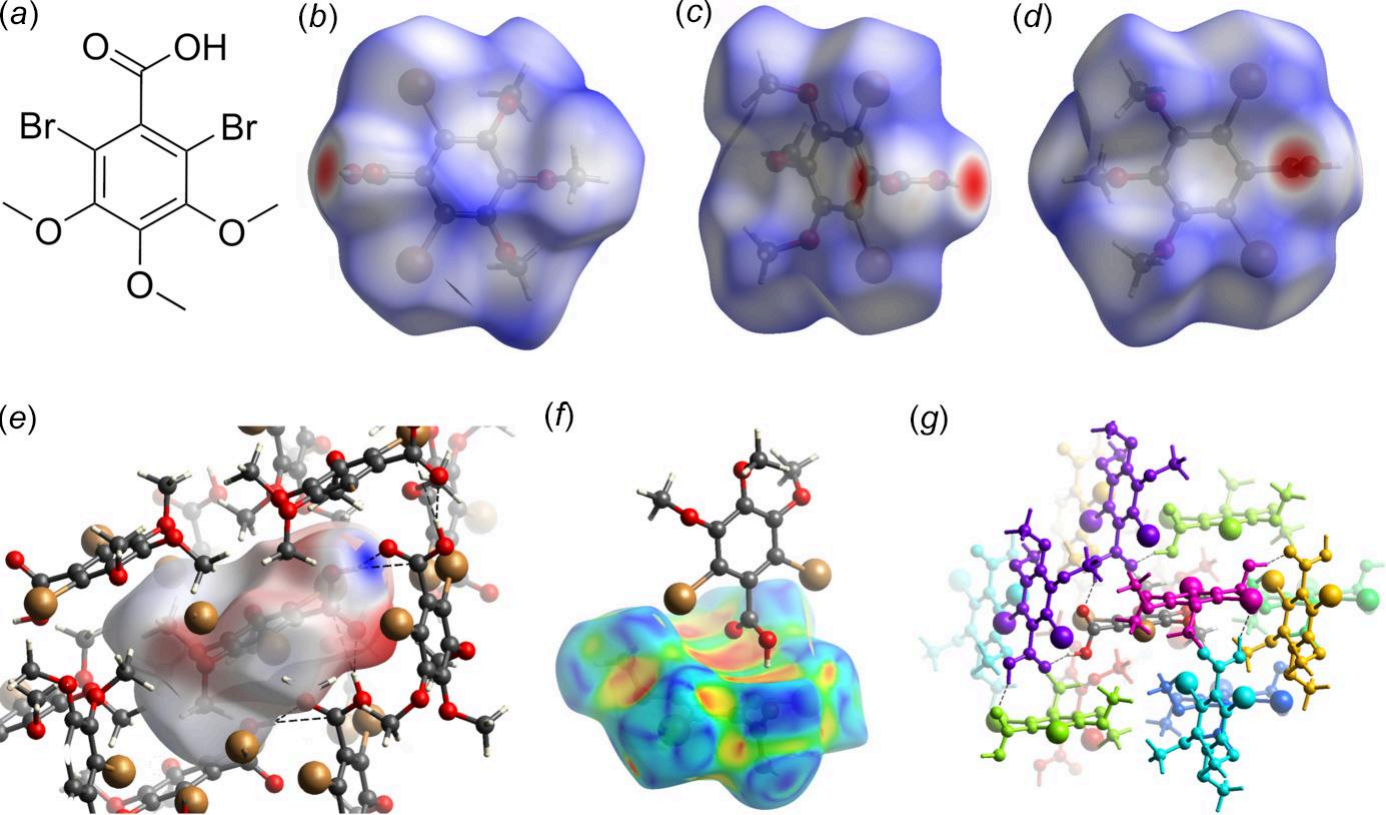
Chemical scheme (*a*) and three different orientations (*b*)–(*d*) of the *d*_norm_ Hirshfeld surface of DBrTMBA. The closest contacts and the eleoctrostatic potential [−0.077, 0.252, (*e*)] at the Hirshfeld surface as well as the curvature of the Hirshfeld surface (f) and overview of the nearest neighbors accompanying Table 2 ▸ (g) are depicted.

To understand the crystal packing of DBrTMBA and the contribution of these closest inter­action contacts, the software program *CrystalExplorer* was used for a Hirshfeld surface and inter­action analysis (Spackman *et al.*, 2021 ▸). Fig. 4 ▸ *b*–*d* show the closest contacts of the hy­droxy­lic acid group as donor/acceptor in hydrogen bonding, as well as the O(lp)–π (C_6_) inter­action in Fig. 4 ▸ *f*. The hydrogen donor/acceptor properties of the carb­oxy­lic group are visualized in the mapping of the electrostatic potential at the Hirshfeld surface (Fig. 4 ▸ *e*).

Table 2 ▸ shows the inter­action energies of DBrTMBA with the closest neighbor mol­ecules in the crystal packing (colors in Fig. 4 ▸ *g*). As expected, the strongest inter­molecular inter­action is exhibited over the carb­oxy­lic hydrogen contacts as well as the O(lp)–π (C_6_) inter­action (purple-coloured neighbors).

**Figure 5:**
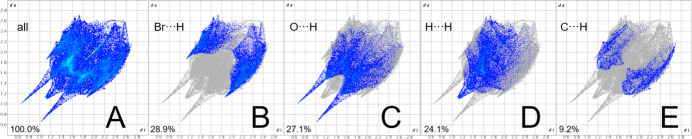
Fingerprint plots of the Hirshfeld surface of DBrTMBA.

The fingerprint plots (Fig. 5 ▸) show the various contributions of Br⋯H, O⋯H, H⋯H and C⋯H inter­actions to the Hirshfeld surface, indicating a high contribution of Br⋯H and O⋯H inter­actions.

## 4. Database survey

Five crystal structures from other authors featuring 3,4,5-tri­meth­oxy­benzoic acid (TMBA) are known in the Cambridge Structural Database (CSD, WebCSD search July 2023; Groom *et al.*, 2016 ▸). The structure of the parent compound, which crystallizes in space group *Pc*, has been reported twice (Qadeer *et al.*, 2007 ▸, Bolte, 2011 ▸). Three other structures contain TMBA co-crystallized with other organic mol­ecules (Thomas *et al.*, 2019 ▸; Chen *et al.*, 2018 ▸; Zhang *et al.*, 2021 ▸). All of them reveal co-planar arrangements of the benzene rings and hydrogen-bonding inter­actions. Furthermore, we recently reported on the previously discussed mono- and di­iodo-3,4,5-tri­meth­oxy­benzoic acids ITMBA and DITMBA (Kolev *et al.*, 2021 ▸, 2023 ▸).

## 5. Synthesis and crystallization

The title compound was synthesized according to the following experimental procedure: A solution of 2-iodo-3,4,5-tri­meth­oxy­benzoic acid (0.36 mmol) in 0.2 *M* NaOH (0.5 mL) was added dropwise to a magnetically stirred aqueous sulfuric acid solution (3.2 *M*, 0.6 mL) of KBrO_3_ (0.72 mmol). The temperature of the reaction mixture was then raised gradually from 294 to 338 K. The resulting solution was stirred for an additional 4.0 h at 338 K and then allowed to cool slowly down (without stirring) to room temperature. The desired product, 2,6-di­bromo-3,4,5-tri­meth­oxy­benzoic acid, crystallized as long, thin needles (m.p. 417–421 K; yield: 30%).

## 6. Refinement

Crystal data, data collection and structure refinement details are summarized in Table 3 ▸. An Hirshfeld Atom Refinement (HAR) using *NoSpherA2* in *Olex2* was performed to obtain non-spherical atomic form factors as well as anisotropic hydrogen atomic displacement parameters (Hirshfeld, 1977 ▸, Kleemiss *et al.*, 2021 ▸). *Orca5* (Neese *et al.*, 2020 ▸) was used for the single-point calculations for the HAR procedure at def2-TZVP/M062X level of theory. The H—*X* distances were fixed to neutron distances from Allen & Bruno (2010 ▸) and refined anisotropically with displacement parameter restraints. The choice to fix the H—*X* distances to neutron distances was made because, even after several attempts at data collection, the data from DBrTMBA did not allow for the refinement of unrestrained hydrogen distances, but did allow for the refinement of softly restrained hydrogen atom anisotropic displacement parameters at these fixed distances.

## Supplementary Material

Crystal structure: contains datablock(s) I. DOI: 10.1107/S2056989023007831/jy2033sup1.cif

Structure factors: contains datablock(s) I. DOI: 10.1107/S2056989023007831/jy2033Isup2.hkl

Supporting information file. DOI: 10.1107/S2056989023007831/jy2033Isup3.cml

CCDC reference: 2281408

Additional supporting information:  crystallographic information; 3D view; checkCIF report

## Figures and Tables

**Table 1:** Hydrogen-bond geometry (Å, °)

*D*—H⋯*A*	*D*—H	H⋯*A*	*D*⋯*A*	*D*—H⋯*A*
O1—H1⋯O2^i^	0.98 (5)	1.68 (3)	2.617 (5)	160 (5)

Symmetry code: (i) 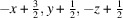 .

**Table 2:** Inter­action Energies (kJ mol^−1^) for the symmetry-generated neighbors of a mol­ecule of DBrTMBA Values calculated with *CrystalExplorer* at the B3LYP/6–31G(d,p) level of theory. el: electrostatic, pol: polarization, disp: energy-dispersive, rep: repulsion.

Color code	Symmetry operation	*E*_el_	*E*_pol_	*E*_disp_	*E*_rep_	*E*_total_
Red	−*x* + 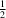 , *y* + 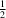 , −*z* + 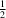	−1.1	−1.0	−14.0	6.1	−10.3
Orange	*x* + 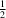 , −*y* + 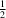 , *z* + 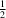	−5.6	−0.7	−10.7	8.3	−10.7
Light green	*x*, *y*, *z*	−6.3	−1.5	−25.8	16.2	−20.2
Green	−*x*, −*y*, −*z*	−3.1	−0.8	−17.3	12.9	−11.0
Cyan	*x* + 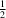 , −*y* + 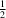 , *z* + 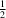	−3.3	−0.3	−7.3	8.7	−4.7
Blue	−*x*, −*y*, −*z*	−9.0	−2.0	37.6	22.1	−30.1
Purple	−*x* + 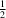 , *y* + 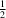 , −*z* + 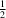	−63.4	−17.1	−28.1	80.0	−54.7
Pink	−*x*, −*y*, −*z*	−1.8	−0.4	−13.8	11.4	−7.1

**Table 3:** Experimental details

Crystal data	
Chemical formula	C_10_H_10_Br_2_O_5_
*M*_r_	370.00
Crystal system, space group	Monoclinic, *P*2_1_/*n*
Temperature (K)	100
*a*, *b*, *c* (Å)	11.4047 (9), 7.1107 (3), 16.8997 (13)
β (°)	107.009 (8)
*V* (Å^3^)	1310.54 (16)
*Z*	4
Radiation type	Mo *K*α
μ (mm^−1^)	6.19
Crystal size (mm)	0.08 × 0.06 × 0.03

Data collection	
Diffractometer	SuperNova, Dualflex, AtlasS2
Absorption correction	Gaussian (*CrysAlis PRO*; Rigaku OD, 2022 ▸)
*T*_min_, *T*_max_	0.749, 0.855
No. of measured, independent and observed [*I* ≥ 2u(*I*)] reflections	18764, 3245, 2254
*R*_int_	0.084
(sin θ/λ)_max_ (Å^−1^)	0.667

Refinement	
*R*[*F* ^2^ > 2σ(*F* ^2^)], *wR*(*F* ^2^), *S*	0.053, 0.123, 1.06
No. of reflections	3245
No. of parameters	218
No. of restraints	63
H-atom treatment	Only H-atom displacement parameters refined
Δρ_max_, Δρ_min_ (e Å^−3^)	1.25, −1.48

Computer programs: *CrysAlis PRO* (Rigaku OD, 2022 ▸), *SHELXT2018/2* (Sheldrick, 2015 ▸), *OLEX2.refine* (Bourhis *et al.*, 2015 ▸), *NoSpherA2* (Kleemiss *et al.*, 2021 ▸), *OLEX2* (Dolomanov *et al.*, 2009 ▸) and *publCIF* (Westrip, 2010 ▸).

## supplementary crystallographic information

### Crystal data

**Table:**

C_10_H_10_Br_2_O_5_	*F*(000) = 718.856
*M**_r_* = 370.00	*D*_x_ = 1.875 Mg m^−^^3^
Monoclinic, *P*2_1_/*n*	Mo *K*α radiation, λ = 0.71073 Å
*a* = 11.4047 (9) Å	Cell parameters from 1911 reflections
*b* = 7.1107 (3) Å	θ = 3.1–28.8°
*c* = 16.8997 (13) Å	µ = 6.19 mm^−^^1^
β = 107.009 (8)°	*T* = 100 K
*V* = 1310.54 (16) Å^3^	Block, clear colourless
*Z* = 4	0.08 × 0.06 × 0.03 mm

### Data collection

**Table:**

SuperNova, Dualflex, AtlasS2 diffractometer	3245 independent reflections
Radiation source: micro-focus sealed X-ray tube, SuperNova (Mo) X-ray Source	2254 reflections with *I*≥ 2u(*I*)
Mirror monochromator	*R*_int_ = 0.084
Detector resolution: 5.2548 pixels mm^-1^	θ_max_ = 28.3°, θ_min_ = 3.1°
ω scans	*h* = −17→19
Absorption correction: gaussian (CrysAlisPro; Rigaku OD, 2022)	*k* = −12→6
*T*_min_ = 0.749, *T*_max_ = 0.855	*l* = −28→28
18764 measured reflections	

### Refinement

**Table:**

Refinement on *F*^2^	4 constraints
Least-squares matrix: full	Primary atom site location: dual
*R*[*F*^2^ > 2σ(*F*^2^)] = 0.053	Only H-atom displacement parameters refined
*wR*(*F*^2^) = 0.123	*w* = 1/[σ^2^(*F*_o_^2^) + (0.0395*P*)^2^ + 3.4625*P*] where *P* = (*F*_o_^2^ + 2*F*_c_^2^)/3
*S* = 1.06	(Δ/σ)_max_ = 0.0002
3245 reflections	Δρ_max_ = 1.25 e Å^−^^3^
218 parameters	Δρ_min_ = −1.48 e Å^−^^3^
63 restraints	

### Fractional atomic coordinates and isotropic or equivalent isotropic displacement parameters (Å^2^)

**Table:**

	*x*	*y*	*z*	*U*_iso_*/*U*_eq_	
Br1	0.86638 (5)	0.67372 (7)	0.45104 (4)	0.03398 (18)	
Br2	0.43161 (5)	0.68854 (7)	0.17096 (3)	0.03341 (18)	
O1	0.6558 (4)	0.9831 (5)	0.3032 (2)	0.0315 (9)	
H1	0.692 (6)	1.0810 (6)	0.277 (4)	0.04 (2)	
O2	0.7570 (4)	0.7934 (5)	0.2410 (3)	0.0384 (11)	
O3	0.7246 (5)	0.3480 (5)	0.5004 (3)	0.0468 (12)	
O4	0.5037 (4)	0.1913 (5)	0.4131 (3)	0.0442 (12)	
O5	0.3643 (4)	0.3576 (5)	0.2669 (3)	0.0484 (13)	
C4	0.6357 (4)	0.6586 (6)	0.3198 (3)	0.0200 (10)	
C5	0.7054 (5)	0.5773 (7)	0.3932 (3)	0.0278 (12)	
C3	0.5234 (5)	0.5822 (7)	0.2773 (4)	0.0284 (12)	
C6	0.6606 (6)	0.4204 (7)	0.4262 (3)	0.0312 (13)	
C2	0.4771 (5)	0.4239 (7)	0.3079 (4)	0.0319 (13)	
C7	0.6884 (5)	0.8184 (7)	0.2838 (3)	0.0261 (12)	
C1	0.5451 (6)	0.3442 (7)	0.3827 (4)	0.0300 (13)	
C8	0.7911 (7)	0.1815 (8)	0.4937 (4)	0.0472 (17)	
H00a	0.852 (3)	0.2105 (16)	0.457 (2)	0.056 (11)	
H00b	0.844 (3)	0.136 (3)	0.5545 (5)	0.057 (10)	
H00c	0.7277 (7)	0.0722 (18)	0.465 (2)	0.048 (10)	
C10	0.3618 (7)	0.1753 (8)	0.2314 (5)	0.0523 (19)	
H00d	0.2696 (10)	0.142 (3)	0.195 (2)	0.066 (11)	
H00e	0.421 (3)	0.173 (2)	0.192 (2)	0.061 (11)	
H00f	0.393 (4)	0.0726 (11)	0.2799 (5)	0.060 (11)	
C9	0.4330 (7)	0.2341 (9)	0.4679 (5)	0.056 (2)	
H00g	0.359 (2)	0.326 (5)	0.4373 (10)	0.062 (10)	
H00h	0.397 (3)	0.1060 (11)	0.485 (2)	0.052 (10)	
H00i	0.4904 (11)	0.302 (5)	0.5224 (12)	0.072 (11)	

### Atomic displacement parameters (Å^2^)

**Table:**

	*U*^11^	*U*^22^	*U*^33^	*U*^12^	*U*^13^	*U*^23^
Br1	0.0308 (3)	0.0207 (3)	0.0382 (4)	0.0033 (2)	−0.0091 (2)	−0.0047 (2)
Br2	0.0382 (4)	0.0181 (3)	0.0312 (3)	0.0032 (2)	−0.0097 (2)	−0.0045 (2)
O1	0.043 (2)	0.0208 (19)	0.038 (2)	−0.0014 (17)	0.0242 (19)	0.0003 (16)
H1	0.08 (4)	0.03 (3)	0.04 (3)	−0.024 (13)	0.033 (17)	−0.006 (13)
O2	0.061 (3)	0.0176 (19)	0.056 (3)	0.0006 (18)	0.047 (2)	0.0001 (18)
O3	0.083 (4)	0.029 (2)	0.031 (2)	0.010 (2)	0.021 (2)	0.0072 (18)
O4	0.068 (3)	0.021 (2)	0.062 (3)	0.0039 (19)	0.048 (3)	0.0028 (19)
O5	0.027 (2)	0.026 (2)	0.089 (4)	−0.0041 (17)	0.013 (2)	−0.007 (2)
C4	0.023 (3)	0.015 (2)	0.025 (3)	−0.0035 (19)	0.012 (2)	−0.0007 (18)
C5	0.035 (3)	0.022 (3)	0.028 (3)	0.004 (2)	0.012 (2)	0.000 (2)
C3	0.026 (3)	0.019 (3)	0.040 (3)	−0.002 (2)	0.009 (2)	−0.002 (2)
C6	0.049 (4)	0.020 (3)	0.028 (3)	0.002 (2)	0.018 (3)	0.004 (2)
C2	0.026 (3)	0.024 (3)	0.050 (4)	−0.005 (2)	0.016 (3)	−0.001 (2)
C7	0.036 (3)	0.014 (2)	0.035 (3)	0.003 (2)	0.022 (2)	−0.001 (2)
C1	0.043 (3)	0.015 (2)	0.040 (3)	−0.001 (2)	0.026 (3)	0.003 (2)
C8	0.068 (5)	0.028 (3)	0.039 (4)	0.009 (3)	0.006 (3)	0.008 (3)
H00a	0.069 (13)	0.05 (3)	0.041 (11)	0.005 (8)	0.006 (6)	0.012 (7)
H00b	0.072 (15)	0.05 (2)	0.042 (7)	0.006 (8)	0.005 (5)	0.014 (5)
H00c	0.066 (17)	0.031 (15)	0.040 (12)	0.011 (8)	0.008 (6)	0.008 (6)
C10	0.050 (5)	0.028 (3)	0.077 (6)	−0.005 (3)	0.016 (4)	−0.009 (3)
H00d	0.054 (7)	0.06 (3)	0.082 (14)	−0.012 (5)	0.015 (5)	−0.003 (8)
H00e	0.053 (10)	0.05 (3)	0.078 (13)	−0.008 (7)	0.016 (6)	−0.010 (8)
H00f	0.063 (13)	0.036 (19)	0.079 (14)	−0.003 (7)	0.018 (6)	−0.007 (8)
C9	0.076 (6)	0.036 (4)	0.081 (6)	0.010 (3)	0.063 (5)	0.010 (4)
H00g	0.082 (12)	0.038 (11)	0.09 (2)	0.012 (5)	0.059 (8)	0.008 (6)
H00h	0.081 (17)	0.033 (9)	0.073 (19)	0.012 (6)	0.070 (8)	0.003 (6)
H00i	0.10 (2)	0.041 (12)	0.092 (12)	0.008 (6)	0.051 (8)	0.007 (6)

### Geometric parameters (Å, º)

**Table:**

Br1—C5	1.935 (6)	C5—C6	1.408 (7)
Br2—C3	1.948 (6)	C3—C2	1.405 (7)
O1—C7	1.299 (6)	C6—C1	1.415 (8)
O2—C7	1.224 (6)	C2—C1	1.395 (8)
O3—C6	1.354 (7)	C8—H00a	1.0770
O3—C8	1.429 (7)	C8—H00b	1.0770
O4—C1	1.346 (6)	C8—H00c	1.0770
O4—C9	1.427 (7)	C10—H00d	1.0770
O5—C2	1.355 (7)	C10—H00e	1.0770
O5—C10	1.425 (7)	C10—H00f	1.0770
C4—C5	1.388 (7)	C9—H00g	1.0770
C4—C3	1.383 (7)	C9—H00h	1.0770
C4—C7	1.496 (7)	C9—H00i	1.0770
			
C1—C2—C3	119.3 (5)	C8—O3—C6	113.4 (5)
C1—C2—O5	121.0 (5)	C9—O4—C1	113.8 (4)
C1—C6—C5	119.2 (5)	H00a—C8—O3	109.5
C1—C6—O3	120.2 (5)	H00b—C8—H00a	109.5
C10—O5—C2	115.5 (5)	H00b—C8—O3	109.5
C2—C1—C6	120.1 (5)	H00c—C8—H00b	109.5
C2—C1—O4	120.6 (6)	H00c—C8—H00a	109.5
C2—C3—C4	120.9 (5)	H00c—C8—O3	109.5
C2—C3—Br2	119.5 (4)	H00d—C10—O5	109.5
C3—C2—O5	119.6 (5)	H00e—C10—H00d	109.5
C3—C4—C5	120.1 (5)	H00e—C10—O5	109.5
C4—C7—O2	122.1 (4)	H00f—C10—H00e	109.5
C4—C7—O1	113.8 (4)	H00f—C10—H00d	109.5
C4—C3—Br2	119.5 (4)	H00f—C10—O5	109.5
C4—C5—Br1	121.0 (4)	H00g—C9—O4	109.5
C5—C6—O3	120.5 (6)	H00h—C9—H00g	109.5
C6—C1—O4	119.3 (5)	H00h—C9—O4	109.5
C6—C5—C4	120.3 (5)	H00i—C9—H00h	109.5
C6—C5—Br1	118.8 (4)	H00i—C9—H00g	109.5
C7—C4—C3	120.5 (5)	H00i—C9—O4	109.5
C7—C4—C5	119.1 (5)	O2—C7—O1	124.0 (5)
C7—O1—H1	109.5		
			
Br1—C5—C4—C3	176.9 (4)	O3—C6—C5—C4	−175.7 (5)
Br1—C5—C4—C7	2.2 (5)	O3—C6—C1—O4	−4.6 (6)
Br1—C5—C6—O3	5.3 (5)	O3—C6—C1—C2	177.3 (5)
Br1—C5—C6—C1	−177.5 (4)	O4—C1—C6—C5	178.3 (5)
Br2—C3—C4—C5	−176.2 (4)	O4—C1—C2—O5	4.4 (6)
Br2—C3—C4—C7	−1.7 (5)	O4—C1—C2—C3	−179.2 (5)
Br2—C3—C2—O5	−5.8 (5)	O5—C2—C3—C4	176.9 (5)
Br2—C3—C2—C1	177.8 (4)	O5—C2—C1—C6	−177.5 (5)
O1—C7—C4—C5	−96.1 (5)	C4—C5—C6—C1	1.4 (6)
O1—C7—C4—C3	89.3 (5)	C4—C3—C2—C1	0.5 (6)
O2—C7—C4—C5	83.0 (6)	C5—C6—C1—C2	0.2 (6)
O2—C7—C4—C3	−91.5 (6)	C3—C2—C1—C6	−1.2 (6)

### Hydrogen-bond geometry (Å, º)

**Table:**

*D*—H···*A*	*D*—H	H···*A*	*D*···*A*	*D*—H···*A*
O1—H1···O2^i^	0.98 (5)	1.68 (3)	2.617 (5)	160 (5)

Symmetry code: (i) −*x*+3/2, *y*+1/2, −*z*+1/2.
